# Age at diagnosis as a prognostic factor in selected categories of juvenile idiopathic arthritis

**DOI:** 10.1136/rmdopen-2024-005369

**Published:** 2025-06-10

**Authors:** Anna-Kaisa Tuomi, Katariina Rebane, Ellen Arnstad, Lillemor Berntson, Anders Fasth, Mia Glerup, Troels Herlin, Hannu Kautiainen, Ellen Berit Nordal, Suvi Peltoniemi, Marite Rygg, Veronika Rypdal, Marek Zak, Kristiina Aalto

**Affiliations:** 1 New Children’s Hospital, Pediatric Research Center, HUS Helsinki University Hospital, Helsinki, Uusimaa, Finland; 2 University of Helsinki, Helsinki, Uusimaa, Finland; 3 Department of Pediatrics, Nord-Trøndelag Hospital Trust, Levanger, Norway; 4 Department of Clinical and Molecular Medicine, Norwegian University of Science and Technology, Trondheim, Trøndelag, Norway; 5 Department of Women’s and Children’s Health, Uppsala Universitet, Uppsala, Sweden; 6 Department of Pediatrics, University of Gothenburg, Gothenburg, Sweden; 7 Institute of Clinical Science, Sahlgrenska Academy, Gothenburg, Sweden; 8 Department of Paediatrics and Adolescent Medicine, Aarhus University Hospital, Aarhus, Denmark; 9 Department of Clinical Medicine, Aarhus Universitet, Aarhus, Denmark; 10 Primary Health Care Unit Kuopio, Kuopio University Hospital, Kuopio, Finland; 11 Folkhälsan Research Center, Helsinki, Finland; 12 Department of Pediatrics and Pediatric Research Group, University Hospital of North Norway, Tromsø, Troms, Norway; 13 Department of Clinical Medicine, Research Group for Child and Adolescent Health, UiT The Arctic University of Norway, Tromsø, Troms, Norway; 14 HUS Inflammation Center, Rheumatology, Helsinki University Central Hospital, Helsinki, Uusimaa, Finland; 15 Department of Pediatrics, St Olavs Hospital Universitetssykehuset i Trondheim, Trondheim, Trøndelag, Norway; 16 Department of Pediatrics, Copenhagen University Hospital, Copenhagen, Denmark

**Keywords:** Arthritis, Juvenile, Health-Related Quality Of Life, Treatment

## Abstract

**Introduction:**

The age at the onset of juvenile idiopathic arthritis (JIA) can influence the trajectory of the disease. We aimed to clarify how age at the visit 6 months after the onset as a continuous variable affects long-term remission of JIA.

**Methods:**

This study investigated 358 patients from the Nordic JIA cohort study. Age at diagnosis was analysed continuously. Three age groups were studied: under 3 years, 3–5 years and over 6 years of age. JIA was categorised as oligoarthritis, seronegative polyarthritis and others (enthesitis-related, psoriatic and undifferentiated arthritis). Clinical data, collected at 6 months after the onset of symptoms, included information about disease activity, uveitis, laboratory test values and medication. The outcomes assessed 17.5 years after diagnosis included remission, health-related quality of life (HRQoL), and functional ability.

**Results:**

The majority of patients with oligoarthritis and polyarthritis were diagnosed before age six, compared with 29% in the group of others. In the oligoarthritis group, predictors of remission included age at diagnosis, male gender, the Juvenile Arthritis Disease Activity Score-71 (JADAS71) and the absence of uveitis. In seronegative polyarthritis, predictors of remission were age at diagnosis and JADAS71 score. In the oligoarthritis group, remission rates were highest in both genders when diagnosed <3 years of age. In the seronegative polyarthritis group, this was not true for female patients. Age at diagnosis had no significant effect on HRQoL or functional ability.

**Conclusions:**

Age at diagnosis in the oligoarthritis was inversely and in the seronegative polyarthritis positively associated with long-term remission in JIA, primarily in females.

**WHAT IS ALREADY KNOWN ON THIS TOPIC**

- There are few studies on the impact of age at diagnosis on the different categories of juvenile idiopathic arthritis (JIA). However, age at disease onset has been studied dichotomously (ie, early vs late disease onset) or has been applied as a mean variable.

**WHAT THIS STUDY ADDS**

- Age at diagnosis, 6 months after the onset, was inversely associated with long-term remission in the oligoarthritis group. In the seronegative polyarthritis group, age at diagnosis was positively associated with long-term remission, particularly in females.

**HOW THIS STUDY MIGHT AFFECT RESEARCH, PRACTICE OR POLICY**

- Our results enable us to identify patients at a higher risk for severe prognosis. This research can also help provide families with more accurate information about the outcomes of JIA. The results may enable us to further tailor the most personalised follow-up strategies. Thus, the findings of this study are beneficial for both families and healthcare professionals.

## Introduction

 Juvenile idiopathic arthritis (JIA) is an autoimmune disease with a chronic course and classified into seven categories.^1^ Of these categories, the most prevalent are oligoarthritis and seronegative polyarthritis.^2^

The outcome of JIA is possibly influenced by gender and age at diagnosis.^3^ The mean age at diagnosis has generally been the focus of several studies on JIA.^4^,^6^ Studies have shown that JIA onset peaks at ages 1–3 years in girls and at ages 2–9 in boys.^4^ In the Nordic JIA study, the highest incidence was observed in girls aged 1–3 years, whereas no peak incidence was identified in boys.^6^ A more recent study from Denmark found no evident peaks for boys at any age at diagnosis, whereas in girls, disease onset peaks before age 5 and at 12–15 years.^7^ Meanwhile, Barnes *et al* analysed gene expression in peripheral blood mononuclear cells and found some differences between early-onset (<6 years) and late-onset (≥6 years) gene expression in various JIA categories.^8^

The effect of age at diagnosis on disease outcomes remains only partially elucidated. Studies have indicated that early-onset JIA is associated with more pronounced joint damage^9 10^ and functional disability^11^ compared with late-onset JIA. A study from the Rheumatic Diseases Portuguese Registry found that a younger age at JIA onset predicted more functional disability, as well as more severe articular and extra-articular damage.^12^ Additionally, earlier disease onset is associated with a lower likelihood of achieving inactive disease in adulthood.^12^ Moreover, girls under the age of 7 years at the time of JIA onset are at risk of developing JIA-related uveitis.^13^ The Nordic JIA study group found that a younger age at JIA onset was associated with JIA-related uveitis in girls but not in boys^14^ and that an earlier age at JIA onset was linked to a higher risk of developing uveitis compared with those with a later onset of JIA.^15^

The clinical outcome of JIA has been the focus of various studies.^316^,^18^ Although different prognostic factors have been proposed, these factors are quite diverse and remain only partially understood.^17 19^ Long-term prognosis is an important concern for parents whose children are newly diagnosed with JIA.^20^ JIA persists into adulthood in more than half of patients,^16 17 21^ necessitating long-term medication,^16 22 23^ which in turn imposes an economic strain on both families and society.^24 25^ Thus, parents are concerned about their children’s prospects concerning JIA and the management of this condition.

In this study, we aimed to elucidate how age at the visit 6 months after the onset as a continuous variable affects the outcome of the various categories of JIA. The primary outcomes were long-term remission including both on or off medication and health-related quality of life (HRQoL) 17.5 years after the diagnosis. We also evaluated secondary outcomes in terms of functional ability 17.5 years after the diagnosis, emphasising the intrusiveness and the burden posed by chronic diseases on families.

## Methods

**Figure 1:**
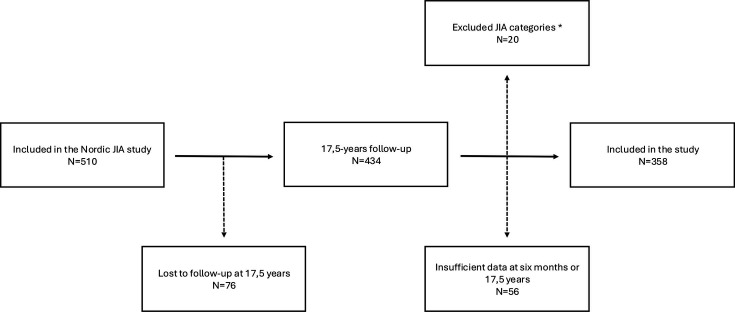
Flow chart of the study population. *Seropositive polyarthritis and systemic-onset. JIA, juvenile idiopathic arthritis.

This study is part of the original population-based Nordic JIA cohort study.^6 26^ Patients newly diagnosed with JIA from specific regions in four Nordic countries (ie, Finland, Sweden, Norway and Denmark) were enrolled consecutively from 1997 to 2000. A total of 510 patients were originally recruited and 434 participated in the 17.5-year follow-up visit. Of these, 358 patients (82%) met the inclusion criteria (figure 1).

For this study, participants were included if clinical data were available 6 months (–1/+2 months) after the onset of disease when diagnosis could be made according to the classification criteria established by the International League of Associations for Rheumatology (ILAR)^2^ and at the 17.5-year follow-up visit. Furthermore, patients with oligoarthritis, seronegative polyarthritis, psoriatic arthritis, enthesitis-related arthritis (ERA) and undifferentiated arthritis were included. The remaining 152 patients from the original Nordic cohort (N=510) were excluded due to failure to attend their 6-month visit (–1/+2 months),^6^ or their 17.5-year visit, or because of insufficient clinical information. Additionally, the categories of seropositive polyarthritis and systemic-onset JIA were excluded, totalling 20 patients.

In this study, we defined age at diagnosis as the time point of JIA categorisation, performed at the visit 6 months after onset of the first symptoms of disease (–1 to + 2 months deviation). In addition, age at diagnosis was considered a continuous variable. Patients were divided into three groups based on age at the diagnosis: (1) under 3 years, (2) 3–5 years and (3) over 6 years. It has been reported that the majority of JIA patients develop symptoms before age 6,^8 27^ and this information served as a criterion for the above grouping. Children under age 6 were further divided into two groups based on their median age.

The patients were further divided into three groups: (1) oligoarthritis group, (2) seronegative polyarthritis group and (3) others (comprising those with ERA, juvenile psoriatic arthritis and undifferentiated arthritis). Children with seropositive polyarthritis (n=6) and systemic-onset JIA (n=14) were excluded due to their limited numbers; in addition, these excluded JIA categories differed from the other JIA categories in terms of both the nature of disease and pathophysiology.^28^ 6 months after disease onset, oligoarthritis is not yet divided into persistent or extended categories according to the ILAR classification.

Clinical data for the present study were collected 6 months after the disease onset and 17.5 years (mean 17.3) after the diagnosis at 6 months. Inflammatory parameters (erythrocyte sedimentation rate and C reactive protein), antinuclear antibodies (ANA), rheumatoid factor (RF) and human leucocyte antigen B27 (HLA-B27) were determined. The cumulative joint count and number of active joints were determined. Disease activity was defined using the Juvenile Arthritis Disease Activity Score based on 71 joints (JADAS71).^29^ Additionally, information regarding the use of medication was collected.

With regard to heredity, we determined whether parents, siblings or grandparents had a history of JIA, uveitis, spondyloarthritis, psoriasis or inflammatory bowel disease.

The provisional Wallace criteria were used to evaluate remission at the 17.5-year follow-up visit.^30^ Remission was defined as inactive disease on medication for 6 continuous months or off medication for 12 continuous months. Remission also included no active uveitis.

The HRQoL at the 17.5-year follow-up visit was evaluated using the 36-Item Short Form Health Survey, which covers eight domains: physical functioning, role limitations due to physical issues, bodily pain, general health perception, vitality, social functioning, role limitations due to emotional issues and mental health. Each domain is scored on a scale of 0–100, with 0 indicating the worst health status and 100 representing the best overall health status.^31 32^ These eight domains are grouped into two major summary scores—mental and physical (PsS and PhS). Scores for the mental and physical domains were standardised using a mean of 50 and an SD of 10,^33^ and functional ability was based on the Health Assessment Questionnaire (HAQ) score.^34^

### Statistics

The summary statistics were described using mean and SD, median and IQR or numbers as percentages. Statistical comparisons between JIA groups were done using analysis of variance (ANOVA), Kruskal-Wallis test and the χ^2^ test. Adjusted (gender and JADAS71 at 6 months) relationship between standardised physical and mental health summary scales 17.5 years after diagnosis according to the age group (<3, 3–5 and ≥6 years) at diagnosis was analysed using two-way ANOVA. Models including main effects (diagnosis and age group) and interaction effects between them. The Sidak multiple comparison procedure was used to correct significance levels for post hoc testing (α 0.05), when appropriate. Multivariate logistic regression analyses were used to identify the appropriate predictors of achieving remission 17.5 years after the diagnosis. The estimated probability of remission 17.5 years after diagnosis as a function of the age at diagnosis was modelled using restricted cubic splines logistic regression models with 3 knots at the 10th, 50th and 90th percentiles. Knot locations were based on Harrell’s recommended percentiles. Models were adjusted for gender and JADAS71 at 6 months. Statistical comparison between genders was made using the Epps-Singleton two-sample test for equality of age distributions. Prediction of reaching remission 17.5 years after the diagnosis with the age at diagnosis ratio was evaluated using AUC (area under the curve), sensitivity, specificity and OR; 95% CIs were obtained by bias-corrected bootstrapping (5000 replications). We defined the best cut-off value using the Liu method, which maximises the product of sensitivity and specificity. In cases of violation of the assumptions (eg, non-normality), a bootstrap method was used for the continuous variables and Monte Carlo p values (small number of observations) were used for the categorical variables. The normality of the variables was evaluated graphically and by using the Shapiro-Wilk W test. The Stata V.18.0 (StataCorp) statistical package was used for the statistical analyses.

## Results

### Characteristics of the study population

Among the 358 patients, 244 (67%) were female, and 120 (33%) were male. The mean age at diagnosis was 6.4 (SD 4.1) years. The oligoarthritis group consisted of 201 patients, the seronegative polyarthritis had 85 patients, and the group of others comprised 72 patients, of whom 25 patients had ERA, 7 had psoriatic arthritis and 40 had undifferentiated arthritis.

### Distribution of JIA patients according to age at diagnosis

**Figure 2:**
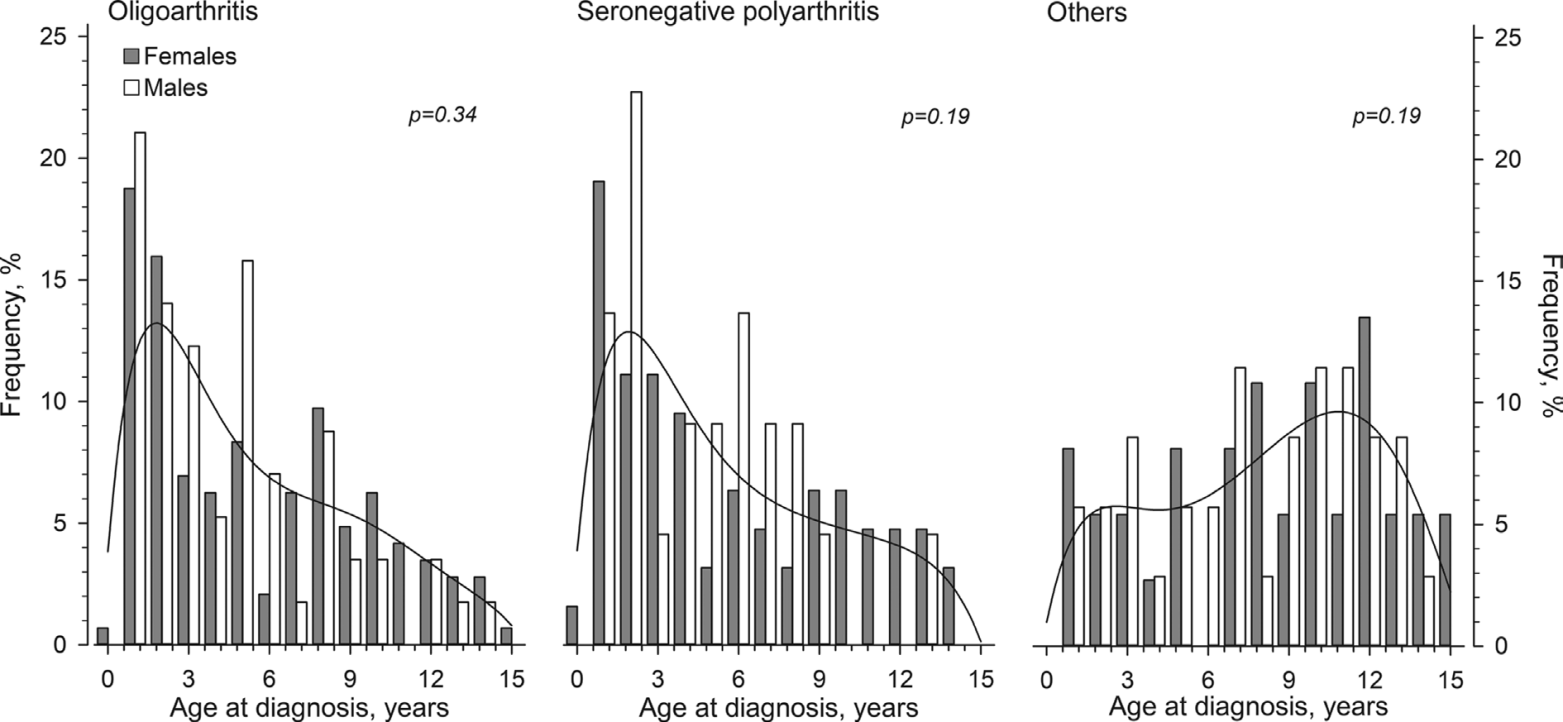
Distribution of age at diagnosis at 6 months after the disease onset according to gender and JIA category. The line represents the local cubic polynomial smoothing for both genders. The p value represents the two-sample test for equality of distributions between genders. JIA, juvenile idiopathic arthritis.

Figure 2 shows the distribution of patients with different JIA categories according to age at diagnosis at 6 months after the disease onset. No statistical difference in age distribution (equality) was observed between the female and male patients in the different groups. In the oligoarthritis group, 60% of the patients were diagnosed before age 6. The proportions of those under age 6 in the seronegative polyarthritis group and in the group of others were 56% and 29%, respectively. Overall, 53% were diagnosed at under 6 years of age. The oldest patients belonged to the group of others (table 1).

**Table 1:** Disease characteristics and clinical data for the assessed JIA categories at 6 months after disease onset

	Oligoarthritis (Ol)N=201	Seronegative Polyarthritis (Po)N=85	Others (Ot)N=72	P value (post hoc)
Female, n (%)	144 (72)	63 (74)	37 (51)	0.002 (Ol/Ot, Po/Ot)
Age at diagnosis, year, mean (SD)	5.8 (3.9)	5.9 (4.0)	8.7 (4.0)	<0.001 (Ol/Ot, Po/Ot)
Uveitis at 6 months from onset, n (%)	20 (11)	7 (8)	4 (6)	0.51
ESR worst 0–6 months from symptom onset, mean (SD)	33 (26)	49 (34)	32 (30)	<0.001 (Ol/Po, Po/Ot)
CRP worst 0–6 months from symptom onset, mean (SD)	18 (28)	36 (45)	26 (49)	0.002 (Ol/Po)
Cumulative joint onset-6 months, median (IQR)	2 (1, 4)	7 (6, 18)	4 (1, 8)	<0.001 (Ol/Po, Po/Ot)
Active joints, n (%)	0 (0, 2)	3 (0, 6)	2 (1, 4)	<0.001 (Ol/Po Ol/Ot)
JADAS71, median (IQR)	0 (0, 2)	3 (0, 6)	2 (1, 4)	<0.001 (Ol/Po, Ol/Ot)
Heredity, n (%)	27 (13)	20 (24)	17 (24)	0.046 (Ol/Po)
ANA positivity, n (%)	97 (51)	40 (49)	16 (23)	<0.001 (Ol/Ot, Po/Ot)
HLA-B27 positivity, n (%)	30 (16)	18 (22)	29 (41)	<0.001 (Ol/Ot, Po/Ot)
Medication ongoing, n (%)				
NSAID	96 (48)	59 (69)	41 (57)	0.003 (Ol/Po)
Sulfasalazine	1 (0)	2 (2)	6 (8)	0.002 (Ol/Ot)
Hydroxychloroquine	7 (4)	6 (7)	1 (1)	0.17
Prednisolone	5 (3)	23 (28)	6 (8)	<0.001 (Ol/Po, Po/Ot)
Methotrexate	10 (5)	29 (35)	6 (8)	<0.001 (Ol/Po, Po/Ot)
Corticosteroid joint injection injections 0–6 months from symptom onset, median (IQR)	1 (0, 2)	3 (0, 6)	1 (0, 2)	<0.001 (Ol/Po, Po/Ot)

Heredity: family history of JIA: uveitis, spondyloarthropathy, psoriasis or inflammatory bowel disease IBD.

* Sidak multiple comparison procedure was used to correct the significance levels for post hoc testing (α=0.05).

ANA, antinuclear antibody; CRP, C reactive protein; ESR, erythrocyte sedimentation rate; HLA-B27, human leucocyte antigen B27; IBD, inflammatory bowel disease; JADAS71, Juvenile Arthritis Disease Activity Score-71; JIA, juvenile idiopathic arthritis; NSAID, non-steroidal anti-inflammatory drugs; Ol, Oligoarthritis; Ot, Others; Po, Seronegative Polyathritis.

### Disease characteristics and clinical data at the time of diagnosis

Table 1 presents the disease characteristics and clinical data of the patients 6 months after disease onset according to JIA categories. Statistical significance was found between age at diagnosis and all of the factors studied, except uveitis and the use of hydroxychloroquine.

### Remission rates for JIA 17.5 years after diagnosis

Remission was observed in 204 patients, with 141 achieving remission. In the oligoarthritis group, 62% (95% CI (54% to 68%)) of patients were in remission, compared with 49% (95% CI (38% to 60%)) in the seronegative polyarthritis group, and 51% (95% CI (39% to 66%)) in the group of others 17.5 years after diagnosis (p=0.012). A significant difference was etected between genders within the oligoarthritis group; the remission rate was higher in males (75% (95% CI 62% to 86%)) than in females (56% (95% CI (48% to 64%)), p=0.012. In the seronegative polyarthritis group, the remission rate was also higher in males (73% (95% CI 50% to 90%)) compared with females (43% (95% CI 30% to 56%)), p=0.016. In the groups of others, no statistical difference in remission rates was observed between females (51% (95% CI 34% to 68%)) and males (51% (95% CI 34% to 69%)).

### Related factors predicting remission of JIA 17.5 years after diagnosis

Table 2 presents the related factors predicting remission at 17.5 years in the two JIA categories. In the oligoarthritis group, age at diagnosis (OR 0.89 (95% CI 0.82 to 0.97)), male gender (2.32 (95% CI 1.09 to 4.98)), JADAS71 score (0.75 (95% CI 0.56 to 1.00)) and uveitis (0.29 (95% CI 0.11 to 0.78)) were found to be associated with the likelihood of remission 17 years after diagnosis at 6 months. In the seronegative polyarthritis group, age at diagnosis (1.15 (95% CI 1.01 to 1.32)) and JADAS71 score (0.82 (95% CI 0.73 to 0.94)) were found to be associated with remission. No related factors were found to be associated with remission in the group of others.

**Table 2:** Multivariate logistic regression analysis for the odds of reaching remission 17.5 years after the diagnosis at 6 months after the disease onset

Measures at the time of 6 months	OligoarthritisOR (95% CI)	Seronegative polyarthritisOR (95% CI)	OthersOR (95% CI)
Age at diagnosis	**0.89 (0.82 to 0.97)**	**1.15 (1.01 to 1.32)**	0.99 (0.86 to 1.14)
Male	**2.32 (1.09 to 4.98)**	3.16 (0.12 to 7.12)	1.00 (0.30 to 3.34)
JADAS71	**0.75 (0.56 to 1.00)**	**0.82 (0.73 to 0.94)**	0.93 (0.82 to 1.07)
Uveitis	**0.29 (0.11 to 0.78)**	0.58 (0.13 to 3.53)	0.20 (0.06 to 2.59)
HLA-B27	0.63 (0.25 to 1.58)	0.23 (0.04 to 1.36)	0.89 (0.26 to 3.01)
ANA positive	1.70 (0.64 to 4.53)	0.91 (0.27 to 3.00)	1.13 (0.32 to 3.95)
Heredity	0.82 (0.42 to 1.61)	0.71 (0.20 to 2.51)	1.58 (0.41 to 6.05)

Heredity: family history of JIA, uveitis, spondyloarthropathy, psoriasis or IBD.

Bold indicates related factors predicting remission

ANA positive, positive antinuclear antibodies; HLA-B27, human leucocyte antigen B27; IBD, inflammatory bowel disease; JADAS71, Juvenile Arthritis Disease Activity Score-71; JIA, juvenile idiopathic arthritis.

### Age at diagnosis and probability of remission 17.5 years after JIA diagnosis at 6 months

**Figure 3:**
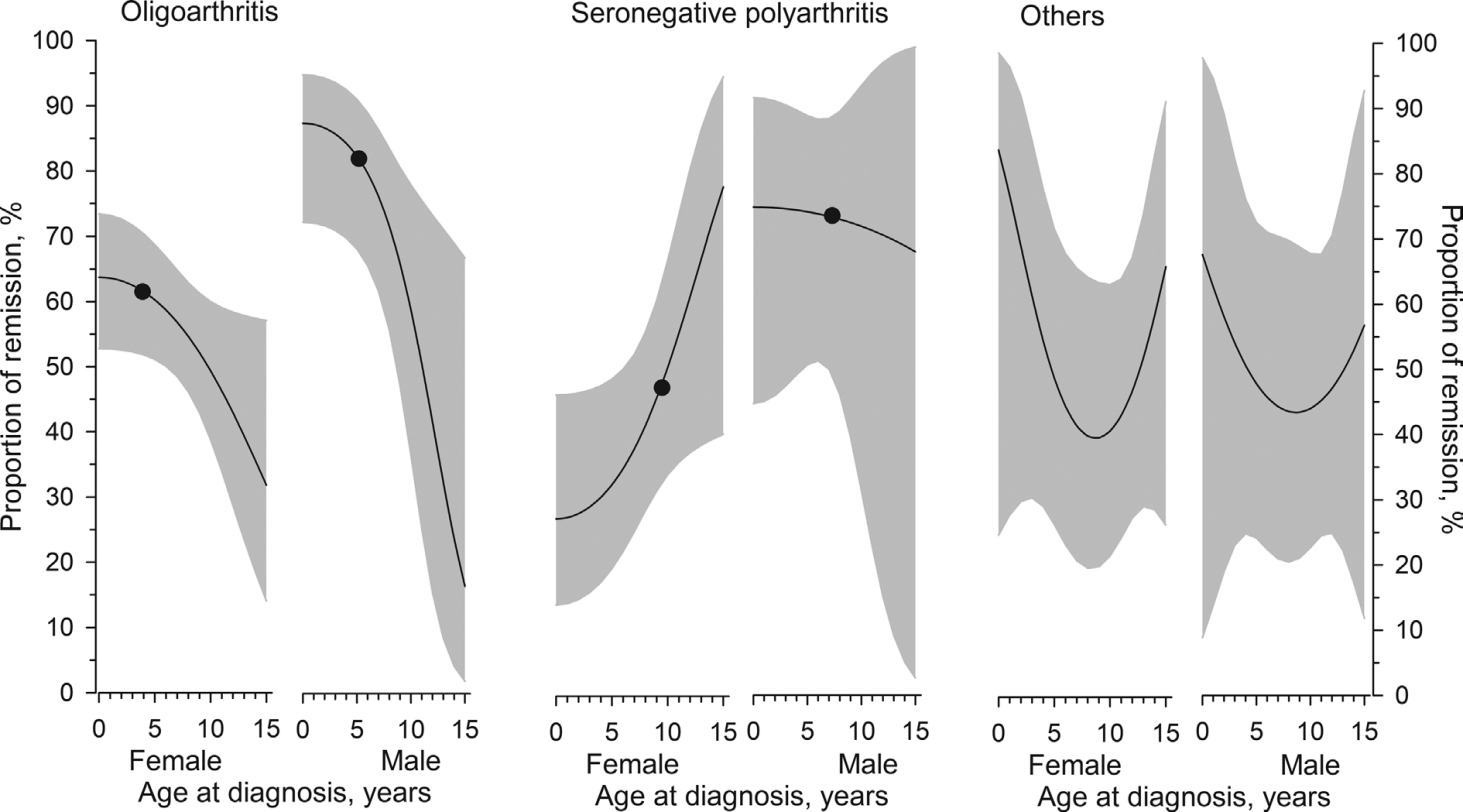
Estimated probability of remission 17.5 years after diagnosis as a function of the age at diagnosis at 6 months after the disease onset. The curves were derived from three-knot restricted cubic splines logistic regression models. The models were adjusted for JADAS71 at 6 months. The grey area represents 95% CIs. The dots represent the optimal cut-off points for age. JADAS71, Juvenile Arthritis Disease Activity Score-71.

Figure 3 shows the relationship between age at diagnosis and remission in different JIA categories adjusted for JADAS71 at 6 months. In both genders in the oligoarthritis group, patients diagnosed with JIA at an earlier age achieved remission at a higher rate after 17.5 years. In the seronegative polyarthritis group, the proportion of female patients in remission after 17.5 years was higher when JIA was diagnosed at an older age. In the group of others, the U-shape curve with an inflection point at 9 years indicated that the proportion of patients in remission after 17.5 years was similar in both genders.

### Cut-off points for age to predict remission 17.5 years after JIA diagnosis at 6 months

Table 3 presents the optimal cut-off points of age for both genders in the three JIA categories. The cut-off points for age that predicted remission in the oligoarthritis group were <3.9 years (AUC 0.58 (95% CI 0.49 to 0.68)) for female patients and <5.2 years (AUC 0.70 (95% CI 0.54 to 0.85)) for male patients. In the seronegative polyarthritis group, the cut-off ages were >9.5 years (AUC 0.60 (95% CI 0.45 to 0.74)) and >5.3 years (AUC 0.58 (95% CI 0.28 to 0.88)) for female and male patients, respectively.

**Table 3:** Prediction of remission 17.5 years after diagnosis at 6 months after the disease onset according to gender and JIA category using the best AUC cut-off points for age

	Optimal cut-off	AUC (95% CI)	Sensitivity (95% CI)	Specificity (95% CI)	OR (95% CI)
All					
Oligoarthritis	<6.1	0.62 (0.53 to 0.70)	0.68 (0.59 to 0.76)	0.52 (0.40 to 0.63)	2.27 (1.27 to 4.06)
Seronegative polyarthritis	>5.3	0.58 (0.45 to 0.70)	0.58 (0.42 to 0.73)	0.67 (0.50 to 0.80)	2.78 (1.16 to 6.66)
Others	<7.1	0.52 (0.39 to 0.66)	0.41 (0.25 to 0.58)	0.77 (0.60 to 0.90)	2.30 (0.84 to 6.30)
Females					
Oligoarthritis	<3.9	0.58 (0.49 to 0.68)	0.49 (0.38 to 0.61)	0.68 (0.55 to 0.79)	2.10 (1.06 to 4.15)
Seronegative polyarthritis	>9.5	0.60 (0.45 to 0.74)	0.62 (0.51 to 0.73)	0.83 (0.67 to 0.94)	3.44 (1.10 to 10.70)
Others	<11.9	0.54 (0.35 to 0.73)	0.79 (0.54 to 0.94)	0.39 (0.17 to 0.64)	2.39 (0.58 to 9.66)
Males					
Oligoarthritis	<5.2	0.70 (0.54 to 0.85)	0.67 (0.53 to 0.81)	0.63 (0.47 to 0.77)	4.22 (1.18 to 14.88)
Seronegative polyarthritis	>5.3	0.58 (0.29 to 0.88)	0.70 (0.49 to 0.90)	0.56 (0.30 to 0.80)	6.43 (0.75 to 12.52)
Others	<7.3	0.50 (0.30 to 0.70)	0.60 (0.45 to 0.76)	0.44 (0.22 to 0.69)	2.60 (0.63 to 10.58)

AUC, area under curve; CI, confidence interval; JIA, juvenile idiopathic arthritis.

### HRQoL 17.5 years after diagnosis at 6 months according to age at diagnosis group

**Figure 4:**
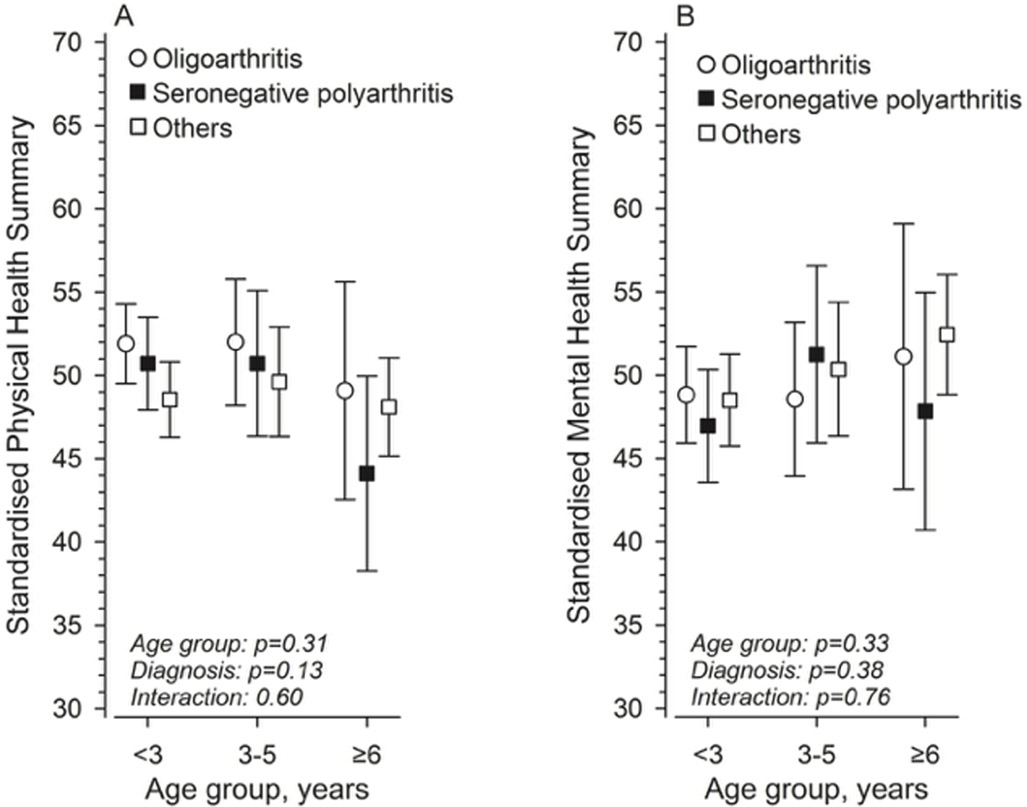
Standardised (A) physical and (B) mental health summary scales 17.5 years after diagnosis at 6 months after the disease according to the age group at diagnosis. The models were adjusted for gender and JADAS71 at 6 months. Whiskers represent the 95% CIs. JADAS71, Juvenile Arthritis Disease Activity Score-71.

Figure 4 shows the HRQoL (physical and mental domains) and age at diagnosis groups 17.5 years after diagnosis, according to the JIA categories, divided into three merged JIA categories. Following adjustment for gender and JADAS71, no significant relationship between age, diagnosis and their interaction was observed.

### Functional ability and disease activity 17.5 years after diagnosis at 6 months according to the arthritis groups at diagnosis

**Figure 5:**
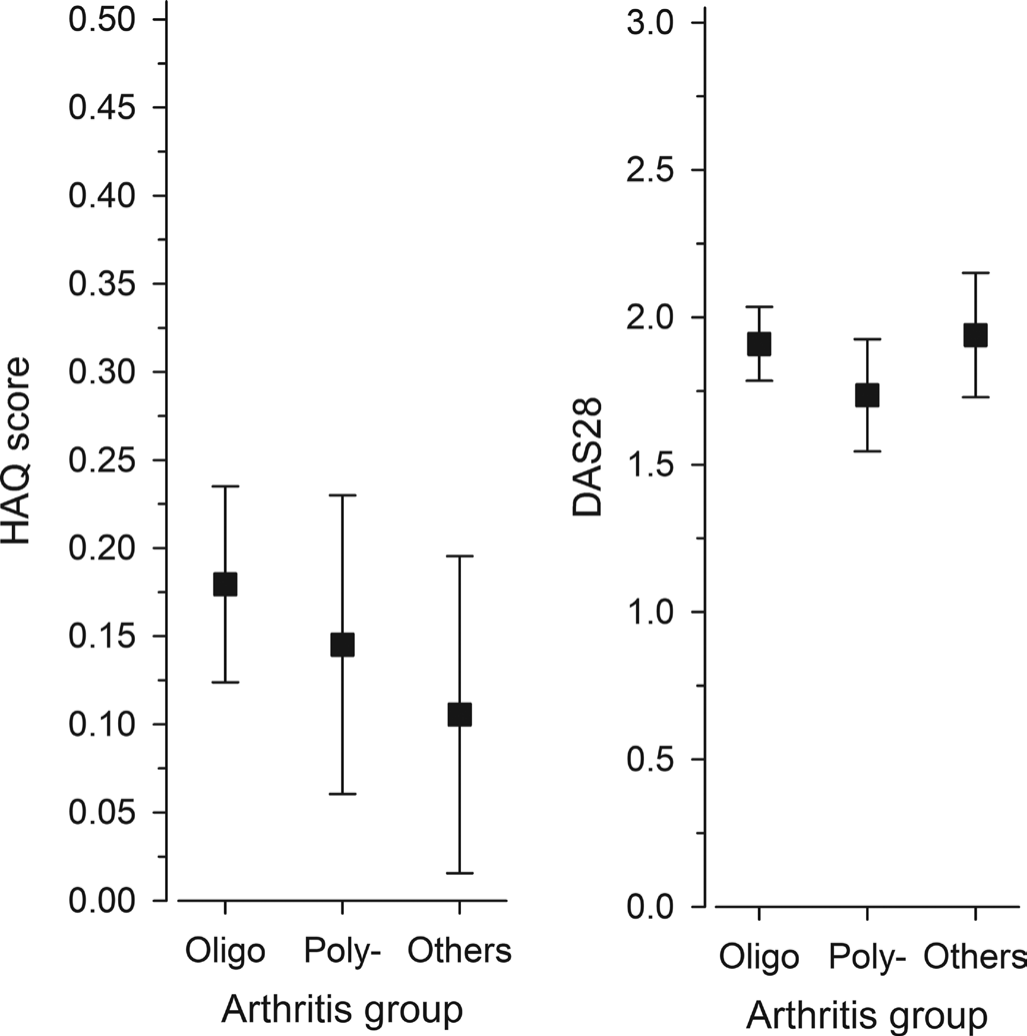
HAQ and DAS28 scores 17.5 years after diagnosis at 6 months after the disease onset according to the arthritis groups at diagnosis. The measures were adjusted for gender and JADAS71 at 6 months. Whiskers represent the 95% CIs. DAS, disease activity score; HAQ, Health Assessment Questionnaire; Ol, oligoarthritis; Po, seronegative polyarthritis; Others, the group of others.

71% of all the patients had no disability based on their HAQ scores. Figure 5 shows the HAQ and DAS28 scores of the investigated JIA groups; the scores did not differ significantly in relation to age at diagnosis as a continuous variable.

## Discussion

Our study demonstrated that, in both genders in the oligoarthritis group, more patients diagnosed with JIA at an earlier age achieved remission, and this was more evident in females. Conversely, in the seronegative polyarthritis group, the proportion of female patients in remission was higher when JIA was diagnosed at an older age. The 6 months related factors predicting remission 17.5 years after diagnosis in the oligoarthritis group included young age, male gender, low JASDAS71 score and absence of uveitis. In the seronegative polyarthritis group, the trend towards remission, especially in female patients, differed from that in the oligoarthritis group. This difference involved gender and the occurrence of uveitis. In the group of others, the probability of remission was U-shaped, indicating that in both genders, remission was mostly achieved by those with either an early or a late age at diagnosis. Early intensive treatment of JIA enhances the possibility of achieving and maintaining remission.^35 36^ Our results may help healthcare professionals and caregivers better understand the significance of age at diagnosis when making shared treatment decisions.

Our primary findings indicated that age at diagnosis as a continuous variable was inversely associated with remission 17.5 years after diagnosis at 6 months in the oligoarthritis group in both genders and positively associated with seronegative polyarthritis, primarily in females. In the seronegative polyarthritis group, older age at diagnosis was positively associated with remission in girls. We then estimated the optimal cut-off points for age. In females, the cut-off points for predicting remission were at a younger age compared with males in the oligoarthritis group. Age at diagnosis was not found to be related to long-term quality of life or functional ability in patients with JIA.

The novel aspect in our study was to investigate the influence of age at diagnosis at the 6 months’ time point as a continuous variable, specifically considering age and gender in JIA and disease outcomes after 17.5 years, focusing on remission and HRQoL. We focused on the independent effect of age at diagnosis as a continuous variable and the outcomes of JIA rather than focusing on the various measures of central age tendency, as in other studies.^4^,^6^ Previously, the Nordic JIA study group reported baseline clinical characteristics as predictors of non-achievement of remission off medication in univariate logistic regression.^19^ No relationship was found between median age at disease onset and remission at an 8-year follow-up.^19^ Similarly, another study from the Nordic JIA study group found that at a follow-up visit of at least 7 years, patients diagnosed at a younger age had a lower rate of achieving remission off medication than those diagnosed at a later age.^22^ This was also found to be independent of the ILAR category.^22^

The unpredictable trajectory of a newly diagnosed chronic disease, the fear surrounding having permanent joint damage or other complications, and the hopes for a healthy life and remission are all significant considerations for the patients, their families and their attending physicians. Parents and caregivers who learn that their child has a chronic rheumatic disease are often very curious about the course and outcome of the disease. This study, which is based on the initial ILAR classification at 6 months, was motivated by these critical concerns, aiming to address the questions and uncertainties families face early in the diagnosis.

Patients with JIA are at risk of social isolation, which has significant consequences on their physical functioning, emotional well-being and educational outcomes.^37^ In our study, we did not find any association between age at diagnosis and quality of life and functional ability. Young adults with JIA were generally doing well; for instance, their education and employment statuses and spousal relationships were comparable to those of the general population.^38^ This information is valuable not only for young adults with JIA but also for parents of children with newly diagnosed JIA.

Uveitis is the most prevalent extra-articular comorbidity related to JIA.^13^ It can be asymptomatic and may have developed prior to the diagnosis of JIA.^39^ The risk factors for uveitis include young age at diagnosis, ANA positivity^14 39^ and oligoarthritis.^39^ In the oligoarthritis group, the absence of uveitis at 6 months was associated with remission 17.5 years after the disease diagnosis. To our knowledge, this association has never been demonstrated in previous studies. Based on our findings, female oligoarthritis patients with uveitis and a high JADAS score on diagnosis conducted at 6 months require closer attention when planning their treatment regimens.

A chronic disease poses a significant burden on patients’ daily lives and affects their overall quality of life. A study from the Nordic JIA study found that 40% of JIA patients had an active disease at an 18 years follow-up^16^ and required direct referral to an adult rheumatology clinic after their paediatric follow-ups ended.^21^ Furthermore, approximately one-third of young adults were not in remission after being symptomless and after suspending medication for a reasonable time before being discharged from a paediatric rheumatology clinic, and thus had to be referred for adult care.^21^ A Finnish study revealed that more than half of the investigated adult patients with JIA had an active disease in their adulthood.^40^

In previous studies, the age at onset has been inversely associated with long-term outcomes in various settings, combining patient populations differently.^12 19 22 41^ One key difference is the grouping of patients by JIA category. Furthermore, our study had a different focus, as we aimed to investigate the association between initial diagnosis and remission.

To our knowledge, the relationships between age as a continuous variable at an early diagnosis and both remission and HRQoL across different JIA categories have not been studied. The strength of this follow-up study is its long-term nature. It includes patients from multiple paediatric rheumatology centres across the Nordic region. Our data also display the richness of data for outcome variables. In total, 82% of the patients originally included in the Nordic JIA study had pertinent information for the analyses in this study.

A potential limitation of the study is the missing data from the 56 excluded patients; however, these data were apparently missing at random. Another possible minor limitation in the study is the heterogeneity of the group of others. We had to exclude patients with seropositive juvenile polyarthritis and systemic onset JIA due to the small number of these patients. This could be considered another limitation of this study.

## Conclusions

Our primary findings indicated that age at diagnosis as a continuous variable was associated with remission 17.5 years after diagnosis at 6 months in both the oligoarthritis and seronegative polyarthritis groups. Our study also demonstrates that the age at diagnosis predicts remission independently as early as 6 months after disease onset, depending on the JIA category and the gender of patients. Given that our study population consists of patients from the Nordic countries, it is reasonable to assume that the results of our study can be generalised to the long-term course of JIA, at least in the context of Nordic patients. Regardless of our findings, more studies on this topic are needed to consider the perspectives of patients and their families.

## Data Availability

Data are available on reasonable request.
